# Using text mining to analyze reflective essays from Japanese medical students after rural community placement

**DOI:** 10.1186/s12909-020-1951-x

**Published:** 2020-02-06

**Authors:** Adam Lebowitz, Kazuhiko Kotani, Yasushi Matsuyama, Masami Matsumura

**Affiliations:** 1 General Studies Department, Jichi Medical University, Tochigi, Japan; 2 Division of Community and Family Medicine, Center for Community Medicine, Jichi Medical University, Tochigi, Japan; 3 Center for Medical Education, Jichi Medical University, Tochigi, Japan; 4 Division of General Internal Medicine, Center for Community Medicine, Jichi Medical University, Tochigi, Japan

**Keywords:** Medical students, Community-based learning, Reflective essays, Learning processes, Text mining, Key word frequency, Regression analysis, Japan

## Abstract

**Background:**

Following community clinical placements, medical students use reflective writing to discover the story of their journey to becoming medical professionals. However, because of assessor bias analyzing these writings qualitatively to generalize learner experiences may be problematic. This study uses a process-oriented text mining approach to better understand meanings of learner experiences by connecting key concepts in extended student reflective essays.

**Methods:**

Text mining quantitative analysis is used on self-evaluative essays (*n* = 47, unique word count range 43–575) by fifth-year students at a regional quota-system university in Japan that specializes in training general practitioners for underserved communities. First, six highly-occurring key words were identified: *patient*, *systemic treatment*, *locale*, *hospital*, *care*, and *training*. Then, standardized keyword frequency analysis robust to overall essay length and keyword volume used individual keywords as “nodes” to calculate per-keyword values for each essay. Finally, Principle Components Analysis and regression were used to analyze key word relationships.

**Results:**

Component loadings were strongest for the keyword *area*, indicating most shared variance. Multiply regressing three of the remaining keywords *hospital*, *systemic treatment*, and *training* yielded R^2^ = 0.45, considered high for this exploratory study. In contrast, direct patient experience for students was difficult to generalize.

**Conclusions:**

Impressions of the practicing area environment were strongest in students, and these impressions were influenced by hospital workplace, treatment provision, and training. Text mining can extract information from larger samples of student essays in an efficient and objective manner, as well as identify patterns between learning situations to create models of the learning experience. Possible implications for community-based clinical learning may be greater understanding of student experiences for on-site precepts benefitting their roles as mentors.

## Background

Reflection and reflective writing in medical education are believed to enhance competency acquisition, and support development of professional identity [1–5]. Writing exercises are useful following clerkships for students to reflect on how they relate to patients [6]. They have been described as a tool to raise empathy in practicing physicians [7]. At the same time, such exercises have been targeted for criticism within the medical community. Some general practitioners view reflection in writing as an ill-defined task that takes time from more important duties [8, 9]. Another argument against these writings is that they have been used invalidly for assessing achievement -- such as attainment of “professionalism” -- removed from their primary function as a tool for cultivating reflection [3, 4, 10]. The current consensus may be that writing is instructive, but *what* it instructs is still not clear [11, 12].

Possibly, the problem lies not in what is being written, but rather how it is being read. That is, qualitative judgment of written reflective narratives may be confounded by reader bias [13]. Triangulation is considered effective in addressing this [13, 14], as are training protocols for “close reading” [5] and for identifying factors related to learner adaptation of educational interventions [15]. However, triangulation is costly [13] and may present difficult challenges in larger data sets. Without rigorous empirical testing of concepts [14] presented in reflective writing, another potential methodological shortcoming to qualitative approaches is the assumption of causative connections between learner experiences [16].

Therefore, assessing medical student experiences in reflective writing may benefit from focused quantitative analyses of essay text and text structure. Here we describe a text-mining method whose goal is to quantify key concepts presented in text as components. These components are based on keywords whose high frequency occurrence and co-occurrence with other keywords are calculated, thereby allowing keyword relationships to be modeled. These models can illustrate learner experiences and augment qualitative methods.

This study, conducted by a regional quota medical program in Japan, employs text mining to analyze student self-evaluation essays following a summer rural placement program called Community-Based Clinical Learning (CBL). In a “quota system”, a set number of slots -- generally 2 to 4 -- is distributed among all 47 prefectures (equivalent to North American states/provinces) for each entering class. In exchange for free tuition at Jichi Medical University (JMU), graduates obligatorily spend 9 years of their early practice in their home prefecture mainly in rural areas [17]. CBL is compulsory four weeks training in clinics and hospitals in such localities for fifth-year clinical students. Following their assignment, students write an essay documenting their schedule and describing their impressions. This study quantitatively analyzed these texts to understand student CBL placement experience.

## Method

Two concepts informed the approach to our analysis. First was statistics-based text mining for its efficacy and relevance for education [18, 19]. Data mining techniques in text analysis can handle large data sets, and so it is useful for investigating writings from larger sample sizes of students. Because statistics specialize in modeling, patterns apparent in sets of written texts can be identified based on data extracted from these texts [18]. Educational process mining [20, 21] is used specifically for this purpose. This subfield of process mining [19, 22] investigates patterns within learning contexts. Initially applied to time-sequence log data to discover patterns towards task completion [23] -- such as acquiring teaching competency of a new theory -- educational process mining is now used to investigate different types of raw data from learning contexts [21]. Once different components within an educational process are identified, the predictive power of components within processes can also be identified empirically.

The next consideration was the central role of *meaning* in a process-oriented approach [24]. Here, meaning centralizes interpretation of experience. In an educational context, meaning translates into *which* learning situations impress students and combine into an overall educational environment. Our technique aimed to identify these situations and measure their most common rate of occurrence within a written narrative. In statistical terminology, this can be called shared common variance.

The CBL student essay is a required submission soon after program completion. The essay is graded by a committee according to three criteria: 1) descriptive detail of experience, 2) explanation of connection to JMU’s mission, and 3) references to research literature. Students are *not* informed of criteria beforehand, nor explicitly advised about essay content or notified of essay scores. However, from first-year coursework, teachers recommend the three following section titles for all community medicine reports:

- Facility and Locale (実習施設と地域概要)
- Training Content (実習内容)
- Thoughts and Observations (考察)

CBL essays automatically follow this convention, necessitating certain keyword appearances from section titles and previous assignments. However, here the final section “Thoughts and Observations” (*kôsatsu*考察) was analyzed for personal impressions of student experience. Compared to the other two, this final section is arguably the least-directed and therefore contains the freest description of learning experiences.

These essay sections first were combined into a single file and analyzed for highly occurring words. Co-occurrence refers to how many times high-frequency words -- i.e., words used often and regarded as “keywords” -- appear in text in proximity to other high-frequency words. Relationships between co-occurring words determine text content. This relationship can be called “strength of inclusion” [25] or more commonly an association [26], and is calculated between “0” and “1” as highest. Co-occurrence network maps visualize how keywords group together throughout an entire text, and connecting lines -- or “edges” -- marked with numerical values indicate association strength, i.e. how often or how closely words occur in text.

However, co-occurrence values do not explain how occurrences between keywords determine occurrence strength with a third keyword. This is known as interaction, and can be determined statistically by calculating how much variance keywords contribute to each other. Calculating variance is useful as it estimates a quantitative percentage “amount” of explanation one keyword receives from one or more other keywords. Looking at this data, word meaning as the writer intends can be plausibly assessed. For example, if word “A” results from the interaction between words “B”, “C”, and “D”, then it is plausible to conclude the writer or group of writers representing a population sample of medical students engaged in community-based learning derives meaning for word “A” from those other words. Clarifying this relationship may be especially important for student reflections on patient relationships. For example, *patient* appears often in community-based service learning program evaluation rubrics and questionnaires [27–29]; however, these formats presuppose types of student experiences with patients and may present a false homogeneity of these experiences [10].

Calculating variance to investigate interaction requires analyzing each text individually; however, text length can skew co-occurrence values, because more keywords have a higher probability of co-occurrence in longer texts. Therefore, investigating interaction requires calculating standardized frequency values within each essay. Highly-occurring words are interpreted as nodes, and proximity of remaining key words determines frequency value.

Frequency value is a standardized measurement, so total word counts and key word counts are removed as confounders, which allows values for all essays to be compared. In addition, outliers can be identified. Thereafter, it is possible to calculate contributions to node words from other keywords -- i.e., how much meaning nodes derive from other words -- in a regression model. Using this model, medical student written evaluations of experiences can be objectively analyzed through highly-occurring keywords by quantifying relationships between these keywords. These relationships plausibly represent the kind of learning occurring in CBL programs.

With the following formula, frequency standardized value z-scores were derived from relationships between each node and remaining five words [30]:

$$ \left(\mathrm{Z}\right)\mathrm{Score}=\left(a-\frac{F_2}{N-{F}_1}\ {F}_1S\right)\div \sqrt{\left(\frac{F_2}{N-{F}_1\ }\right)\ \left(1-\frac{F_2}{N-{F}_1}\right)} $$

F_1_ = Frequency of Node Word in entire data.

F_2_ = Frequency of Word W in entire data.

a = Frequency of Word W before and after Node Word within Width of Window Span, i.e., word proximity distance from Node Word.

S = Width of Window Span = 10 (Five words to the Left + Five words to the Right).

*N* = Total text word count.

Texts were analyzed by KH Coder 3.0 (http://khcoder.net/en/index.html), a free downloadable multilingual text-mining program developed by Koichi Higuchi, Ritsumeikan University, Japan. Statistical analysis was done on SPSS software version 23 for Windows (IBM Japan Inc., Tokyo, Japan). This study was approved by the Jichi Medical University Ethical Committee (approval #18–047), and complies with informed voluntariness.

## Results

CBL essays are annually collected and distributed on DVD to university faculty. E-mails were sent to 131 fifth-year students requesting approval to use their essay assignment for this study. Forty-nine responded positively (37%). Essays record daily clinical experiences and conclude with a personal “Thoughts and Observations” (*kôsatsu*考察) section. When these sections were uploaded to KH Coder for analysis, software could not read one essay’s text, so in total 48 essays were initially tested. Total word count ranged from 158 to 3067 (*M* = 1027, Median = 796, SD = 716); unique words per essay ranged from 43 to 575, *M* = 219, Median = 184, SD = 126. Word counts were equally distributed with no outliers.

First, a brute-force word extraction of combined essays (49,293 words total, 10,519 unique terms) discovered six noun terms occurring most frequently:

1. patient (*kanja*患者): 402
2. systemic treatment (*iryô*医療): 359
3. locale (*chi’iki*地域): 347
4. hospital (*byôin*病院): 318
5. care (*shinryô*診療): 274
6. training (*jisshû*実習): 207

**Fig. 1:**
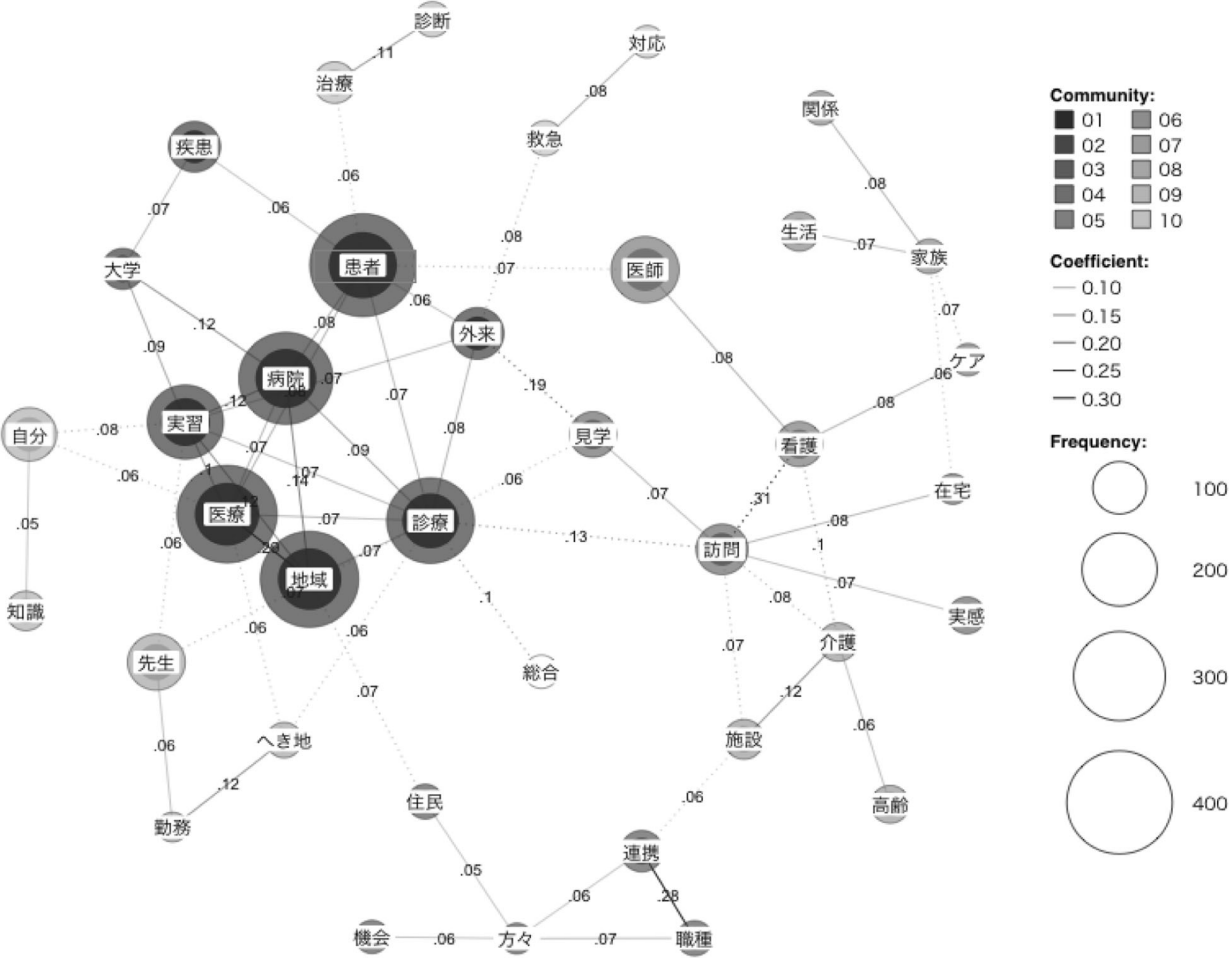
Co-occurrence network map with degree values, with six most frequently-occurring terms clustering together

Both *iryô* and *shinryô* are modes of treatment. *Iryô* is systematically provided by a clinic, health care organization, or administrative area; *shinryô* is provided personally by a physician, and to avoid confusion was translated as *care* for this analysis. These were the six most frequently appearing nouns in the data sample, and were followed by the verbs *think* and *feel*. The verbs were considered too general to reveal useful data, as was the next occurring noun *doctor* (164 times). Table 1 shows degrees of co-occurrence between each key word, and Fig. 1 illustrates word grouping.

**Table 1:** Degrees of co-occurrence between keywords based on keyword map (empty cells: <.05)

	patient	treatment	locale	hospital	care	training
patient						
treatment	.08					
locale		.29				
hospital	.08	.07	.14			
care	.07	.07	.07	.09		
training		.10	.12	.07	.07

Next, proximal to each keyword as node, the above z-score formula calculated standardized frequency values for remaining keyword terms. Then, each node’s z-scores were summed to create node word variables. For example, the following were used for keyword *patient* as a node word:

(patient.node.systemictreatment.z) + (patient.node.locale.z) + (patient.node.hospital.z) + (patient.node.care.z) + (patient.node.training.z) = patient.node.z.sum.

Finally, all nodes were summed:

patient.node.z.sum + systemictreatment.node.z.sum + locale.node.z.sum + hospital.node.z.sum + care.node.z.sum + training.node.z.sum = node.z.sum.total.

This provided each essay with a single value representing both presence and strength of keyword terms. Strength refers to the degree of connection one keyword had with remaining ones. Sum total node values were then tested for regular distribution, and one outlier was identified and removed. This left *n* = 47 essays for analysis with the following descriptive data (Table 2):

**Table 2:** Descriptive data of standardized summed keyword values

	Mean	Median	SD	Range	Minimum	Maximum
patient.node.z.sum	0.35	0.00	0.92	5.33	−1.86	3.47
treatment.node.z.sum	3.38	3.07	2.71	9.80	0.00	9.80
locale.node.z.sum	3.96	3.63	2.96	10.73	0.00	10.73
hospital.node.z.sum	2.17	2.21	2.25	11.74	−2.11	9.63
care.node.z.sum	1.18	0.54	1.65	7.79	−0.18	7.61
training.node.z.sum	1.70	0.83	2.34	12.58	−0.15	12.43
node.z.sum.total	12.75	13.26	7.73	34.47	0.00	34.47

Because Extracted Words*z.sum and Unique Words*z.sum *r*-values for each individual node are similar (Table 3), Pearson correlations suggest convergent and internal validity of standardized frequency values measured as variables.

**Table 3:** Pearson Correlations between summed frequency values of key words as nodes **p < 0.01, *p < 0.05 (2-tailed)

		Extracted Words	Unique Words	node.z.sum						
				patient	treatment	locale	hospital	care	training	total
Extracted Words		1								
Unique Words		.99**	1							
node.z.sum	*patient*	.04	.08	1						
	*treatment*	.23	.21	.09	1					
	*locale*	.31*	.31*	.06	.45**	1				
	*hospital*	−.04	−.05	−.02	−.13	.43**	1			
	*care*	.06	.04	.22	−.12	.15	.33*	1		
	*training*	−.10	−.11	.21	.27	.30*	.39**	.17	1	
	total	.18	.16	.28	.55**	.80**	.60**	.40**	.69**	1

Next, a Principle Components Analysis (PCA) was conducted to understand amount of shared variances. Technically, PCA reduces numbers of items to create components based on shared variances. Item reduction is not this study goal, but result data included common variances among standardized summed values of keywords as node words within each student essay. Based on these variances, it becomes possible to create a regression model showing how certain keywords predict others, interpretable as quantifiable meaning contributed between certain keywords. These keyword relationships can help assessors evaluate student learning experiences. Extracted components with eigenvalues over 1.0 explained 74.16% of total variance (Component 1: 33.86%, Component 2: 22.35%, Component 3: 17.95%). The pattern matrix (Table 4) shows *systemic treatment*, *hospital*, *locale*, and *training* loading together with *locale* loading highest (.835).

**Table 4:** Pattern Matrix for Principle Components Analysis for standardized frequency values of key words (Rotation Method: Oblimin with Kaiser Normalization)

	1	2	3
systemic treatment.node.z.sum	0.398	0.819	0.136
care.node.z.sum	0.29	−0.589	0.385
patient.node.z.sum	−0.087	0.013	0.943
hospital.node.z.sum	0.764	−0.5	− 0.198
locale.node.z.sum	**0.835**	0.259	−0.096
training.node.z.sum	0.631	0.063	0.264

When deciding which factors fit into a regression model based on standardized pattern matrix data, loading sizes are compared to probable significant effect sizes. These would likely be equal to or higher than .40 with this sample size. These results indicate *locale* variance in common with other node z-values except for *care and patient*. Therefore, predictive value for node keyword *locale* from three remaining high-loading keyword node items was examined. The regression model showed keywords *systemic treatment*, *hospital*, and *training* predicted 45% of keyword *locale* variance (*R*^2^ = .45, *p* < .0001). Furthermore, the high factor loading of this keyword is congruent to *locale*’s high co-occurrence degree discussed earlier, suggesting internal and convergent validity for this method.

In contrast, the node keyword *patient* seemed to inhabit its own component independently and added the least amount of variance overall. In this third component, node keyword *care* loaded the next highest. As stated earlier, *care* refers to physician-provided personal treatment, which plausibly connected to student experiences with patients and suggests a conceptual relationship between keywords. Although keyword node *care*’s prediction level for keyword node *patient* was too low for significance (*R*^2^ = .047, *p* = .142), its highest loading among other factors for *patient* suggested construct validity for our methodology.

## Discussion

In this study, student reflective essays were examined by identifying keyword terms by volume, as well as keyword term relationships analyzed by standardizing frequencies. Using standardized z-measurements for frequencies allowed data comparisons between essays of different length and keyword amounts. Frequencies revealed keyword “strengths” in quantifiable values as nodes via proximity to other words. Models calculated with node strengths as variables provide insight into student clinical community placement experiences, with high keyword occurrence and frequency to other keywords as a proxy measurement for experiences within this learning environment. Data extracted from reflective essays suggests student experiences were most strongly informed by locale of clinical practice, and locale experiences were informed by hospital, training, and systemic medical treatment experiences. Stated as process, locale’s meaning derived from these other three learning situations. Calculated as components within a statistical model, almost 50% of keyword *locale*’s variance was shared by *hospital*, *training*, and *systemic medical treatment*. Because data came from text analysis of freely written essays and not from more definitive markers such as survey items with proven psychometric properties, we consider this result high. Furthermore, results interpreted as four-way analyses of variance (ANOVA) -- F (46) = 8.78, *p* < .001 -- suggest interaction increases predictive power.

In contrast, although keyword *patient* had the highest frequency on aggregate among all essays, its relationship with other high-frequency node words was inconsistent save for a weak relationship with keyword *care*. This keyword’s low associations with other node keywords suggest patient experiences important but highly individualized for each student, and therefore not easily generalizable from the perspective of the educational process. These results are notable contextualized against other studies of community-based service learning programs using evaluation rubrics and questionnaires [27–29]. Overall, it suggests general conclusions about student-patient experiences in clinical placements may be elusive, and standardized survey instrument validity may be problematic.

Results here also suggest certain properties for the degrees of co-occurrence metric. Co-occurrence is essentially an association value. However, as with a correlation measure, precise quantification is difficult; i.e., Mane and Börner state “words that appear together often will have a strength closer to 1, and words that never appear together, a strength of 0” (24:5288), but without clarifying how “often” can be precisely interpreted as a standardized measurement. This study’s method overcomes this problem. Interestingly, degrees of co-occurrence were highest between other keywords and *locale* (*systemic treatment = .29, *hospital = .14, *training = .12). This suggests co-occurrence as a calculation may have common properties with shared variance.

Some methodological shortcomings should be noted. One involves approach: specifically, could the *lack* of association between highly-occurring words in fact be more significant? This line of inquiry could be the subject of a future study, and is certainly justifiable since there is still a lack of consensus about how complex ideas regarding professionalism can be best taught in community settings [31]. In addition, as this study is exploratory authors did not predetermine term number, nor cut-off for instances of occurrence. Overall, whether our method accurately represents meanings or themes through quantifiable relationships will ultimately depend on larger data samples and replication.

Another possible shortcoming is these essays are distributed to university faculty, so students might positively bias self-evaluation by downplaying difficulties faced during CBL. To consider this, frequencies of negative descriptors *difficult*, *problem* and *unusual* were investigated and found to occur less frequently (30–40 times) than other descriptors denoting *importance* or *necessity* (45–110). Interestingly, *satisfaction* does not appear. More revealingly, *relation/relationship* appeared 42 times but its z-frequency proximity to *patient* was low at .834 on a scale ranging from 26.433 to − 1.151 for other unique words. Rhetorical and linguistic differences aside, data here questions general assumptions of how students “know” patients as reported in previous studies about community-based service learning programs [27–29]. Furthermore, even if self-perceived patient knowledge is believed positive, predictive validity for future performance has not been shown [32, 33].

## Conclusion

In exchange for free tuition, Jichi Medical University graduates are required to practice in potentially underserved locations. Summer placements are an essential part of the curriculum, and reflective essays are an important data source for understanding the educational process in terms of which experiences have significant meanings for students. Text mining can extract information from larger samples of student essays in an efficient and objective manner, and the technique described here can identify patterns between learning situations to create models of the entire learning experience. Results from this method can converge with results from traditional qualitative methods to raise data validity and better illuminate a medical student’s journey to professionalism.

## Data Availability

The datasets used and/or analyzed during the current study are available from the corresponding author on reasonable request.

## Abbreviations

ANOVA: Analyses of Variance
CBL: Community-Based Clinical Learning
JMU: Jichi Medical University
PCA: Principle Components Analysis
